# The color of risk protection orders: gun violence, gun laws, and racial justice

**DOI:** 10.1186/s40621-020-00272-z

**Published:** 2020-08-10

**Authors:** Jeffrey W. Swanson

**Affiliations:** 1 Department of Psychiatry and Behavioral Sciences, Duke University School of Medicine, Box 3071 Med Ctr, Durham, NC 27710 USA; 2 Brightleaf Square, Suite 23E, 905 W. Main St, Durham, NC USA

**Keywords:** Extreme risk protection orders, Gun violence, Gun laws, Gun rights, Firearm injury and prevention, Racial disparities, Racial justice

## Abstract

**Background:**

Extreme risk protection order (ERPO) laws give municipal police officers new authority, through a civil restraining order, to remove firearms from people who are deemed to pose an imminent risk of causing serious harm to others or themselves. Despite the expected public safety benefit of ERPOs, it is possible that implicit racial bias could infect ERPO petitions, the court processes that authorize and extend the gun removal orders, or the behavior of the police in serving and enforcing them. How might potential racial disparities in ERPO implementation qualify the evidence that this intervention saves lives? What should gun violence prevention researchers and stakeholders do to promote racial justice?

**Main text:**

This commentary reflects upon an innovative and promising legal tool for gun violence prevention through the lens of racial justice concerns. Emerging research, guided by a public health paradigm, asks whether ERPOs save lives. But now is the time to pose other important questions as well. Preventing gun violence and mitigating the disproportionate impact of arrests and incarceration in communities of color are two important goals that may collide. The origin story of many U.S. firearm restrictions, and the continuing disparities in their enforcement and implementation, are intertwined with the legacy of systemic racial discrimination in policing and criminal justice in the United States. The public policy challenge of balancing risk and rights is increasingly fraught, especially as constituencies with a different interest in one or the other concern find themselves standing on opposite sides of a privilege chasm.

**Conclusion:**

Saving lives from gun violence matters, but ensuring that the lives saved are also respected--free from racial oppression, afforded equal justice--also matters. This commentary is a call to include racial disparities impact as an essential outcome of interest for ERPO studies specifically, but more broadly for all public health law research studies at the intersection of firearm injury prevention, law enforcement, and criminal justice.

## Introduction

Firearm injury scholars have long looked to the larger discipline of public health--its familiar conceptual architecture and scientific research toolkit--to frame the American gun violence “epidemic,” measure its reach, and evaluate policy solutions to address it (Hemenway 2017; Webster and Vernick 2013). The public health paradigm has tended to reduce the complex social pathologies around gun violence to the universal metric of mortality; what has mattered most is simply the number of human deaths caused by firearm injuries, and the number of lives to be saved through public health interventions, policies and laws (Bauchner et al. 2017). In 2020, things are looking different for public health in the United States, in ways that may call into question the mainstream assumptions and priorities of gun violence prevention scholarship and policy development.

The entire field of public health is now preoccupied to some degree with an global pandemic that seems to have taken deliberate aim at communities of color. The reasons for this are numerous and complex, but they implicate social determinants of health and illness, illuminate glaring disparities in our systems of care, and indict our fractured and hollowed-out governance structures for the paralysis and chaos of our public health response (Metzl et al. 2020). At the same time, an emerging social movement in reaction to the killing of George Floyd--yet another unarmed black American whose final pleas for mercy went unheeded by yet another white police officer--now invite the field of public health to reexamine its intellectual enterprise and surrounding institutional matrices through the lens of racial justice. Gun violence prevention research and policy must join in that reckoning.

Beyond mere reflection and recognition of the nation’s legacy of injustice, real action is required. To start with, our field needs to think of reducing racial disparities as an essential public health outcome to be studied in all evaluations of injury prevention and public safety policies, but perhaps especially those at the intersection of gun violence prevention, law enforcement, and criminal justice (Fliss et al. 2020).

Of late, gun violence prevention researchers have set about to examine evidence for the effectiveness of an innovative legal tool that gives municipal police officers new authority, through a civil restraining order, to remove firearms from people who are behaving dangerously (Swanson 2019). Apropos of the public health model, again, the driving policy question has been whether this tool--an extreme risk protection order (ERPO)--ultimately saves lives. But now comes a moment to ask difficult questions about its deployment: Is this new tool in the hands of police being used fairly? Could ERPOs even bring harm to some groups of people, perhaps indirectly, in ways that the policy’s well-meaning legal architects had not anticipated? An imperative for implementing the law, along with every other intervention to keep firearms away from people at risk of harming others or themselves, must be to ensure respect for the rights, dignity, and wellbeing of those who, for far too long, have lived under the crushing weight of racial discrimination.

## Extreme risk protection orders and race

This issue of *Injury Epidemiology* features a new research report on the implementation of extreme risk protection orders (ERPOs) in King County, Washington, which includes the city of Seattle (Frattaroli et al. 2020). An easily overlooked finding in this small study now stands out in bold relief: black people were overrepresented in gun removal orders by a factor of nearly 2 to 1 compared to their share of the county population (12.0% vs. 6.9%). Intending no irony, the authors comment on this difference by invoking racial equivalence: *“We do note that both white and black respondents are over-represented in the King County data* (Frattaroli et al. 2020).” In blunt statistical terms they are correct: there is a higher proportion of white people, too, among ERPO respondents than in the county population: 74.7% vs. 66.9%, with Latinx whites folded in as white. But do these differences mean the same thing for black and white respondents? Probably not.

The King County study did not report separately for racial subgroups any other characteristics of ERPO respondents, the “dangerous behaviors” by which they were deemed to meet the standard for gun removal, or the features of the ERPO petitions and court process. The study sample size was probably too small to render any statistically informative analyses of potential differences or disparities by race in the specific parameters of ERPOs. But we should not ignore the fact that virtually all of these civil restraining orders were initiated by law enforcement officers, and approved by judges, who are embedded in systems of criminal justice that for decades have ensnared young men of color in staggeringly disproportionate numbers (Alexander 2010; Thompson 2019). Similarly, police officers were the petitioners in 97% of ERPO cases in California, a recent report shows (Pallin et al. 2020). Thus, if the statutory criteria for ERPOs included criminal history, one might expect upstream racial imbalances in law enforcement and the justice system to be reproduced in the demographics of ERPO respondents. (Gun restrictions in general--for example, the disqualification of convicted felons from purchasing or possessing firearms--have been subject to critical deconstruction as racist in their effects, insofar as convicted individuals may have been unfairly pre-selected for gun disqualification by a racially discriminatory justice system.) ERPOs are not formally linked to criminal records, of course, but the King County study reports that 37% of ERPO respondents indeed had a criminal history. Given that fact, and the role of the human perception of “risk” in the whole process, is it possible that implicit racial bias could infect ERPO petitions, the court’s decisions to grant them, or the behavior of the police in carrying them out? If that turned out to be true, how might it recast the evidence that ERPOs save lives?

The authors mention the need to know more about the use of ERPOs in *“… communities of color where relationships with law enforcement are too often strained* (Frattaroli et al. 2020).” But they downplay the potential for negative impact of ERPOs by noting the lack of “collateral consequences” such as criminal arrest or involuntary treatment. Such consequences are not always avoided. Our recent study of Indiana’s experience with a risk-based firearm removal law found that 1 in 5 respondents were arrested, either in conjunction with the firearm seizure event or during the subsequent 12 months covered by the order; 17% of those arrests resulted in charges for firearm offenses--behavior that may not even have been illegal absent the ERPO order (Swanson et al. 2019). While ERPOs are not criminalizing per se, noncompliance with them can be; this creates an avenue by which unequal enforcement along racial lines could perpetuate justice disparities.

The Indiana study provides an instructive example of the need to look beneath the surface of anodyne results. In our initial analysis, the question of whether the state’s gun seizure law could have disproportionately targeted racial minorities ended with the simple finding that nonwhites were underrepresented among gun removal cases in comparison to their share of the Marion County population (24% vs. 38%, respectively). However, a subsequent lookback at the court process and outcomes tells a subtly different story. We discovered that a significantly higher percentage of nonwhite than white individuals failed to appear at a scheduled court hearing to seek the return of their guns, and thus lost their guns by default (63% vs. 51%; *p* < 0.05). Regarding outcomes following gun removal, 14 individuals from the white subgroup went on to die of suicide, which equates to an annualized suicide rate 31 times higher than the average age-adjusted suicide rate in the general adult population of Indiana during the same time period (Swanson et al. 2019). Among the nonwhite subgroup in the study, not a single person died of suicide.

These new analyses provoke new questions: Does the finding of racial difference in the rate of failure-to-appear reflect autonomous individual choices, alienation from law enforcement and suspicion of courts, or systemic barriers to justice? Does the difference by race in suicide outcomes merely reflect a pattern in the population suicide rate (higher for whites overall), or did the law in practice select nonwhite individuals for gun seizure using a different standard or lower threshold of risk than their white counterparts? We do not know. The patterns in the data could easily have resulted from other baseline characteristics and features of the cases that were correlated with race but opaque to our analysis. It is also possible that implicit racial bias played at least some role, for example, in various actors’ perceptions of the type of risk or behavior meriting gun removal, the process of referral to the intervention, and in the responses of the police and judges; any of these factors might have contributed to some significant selection or different treatment of gun seizure candidates along racial lines. In any case, the backstory of other gun restriction regimes in the United States should give us reason to inquire.

## Racial injustice, the origin story of gun laws, and contemporary echoes

The history of gun control laws in the United States (as with the history of every other social institution in this country) is intertwined with a legacy of white supremacy and racial injustice (Cottrol and Diamond 1994). Considering just one example, North Carolina’s pistol permit requirement was enacted in 1919 with the provision that a permit could be denied to anyone who a county sheriff deemed to lack “good moral character.” Peering backward through the lens of twenty-first century sensibilities, one can hardly fail to see the potentially racist impact, if not the explicit intent of such a law in the Jim Crow South. In the words of a lawmaker from the neighboring state of Tennessee during the same era: *“Here we have laid bare the principal cause for the high murder rate in Memphis--the carrying by colored people of a concealed deadly weapon, most often a pistol. Can we not cope with this situation?* (Cramer 2016)*”.*

A century later, national surveys still find a substantial difference between racial groups in gun ownership and access, with about half of white respondents but only one third of black respondents reporting gun access at home (Parker et al. 2017). There could be many reasons for this, but the historically disparate application of laws like North Carolina’s pistol permit requirement seems plausibly to have been a contributing factor. Given the ignoble legislative origin story of many gun laws and that story’s modern echoes, how should public health scholars and stakeholders in the gun debate (Cook and Goss 2020) think about these restrictions today? Is the legal practice of risk-based gun removal to be suspected of prejudice, too, if it is directed disproportionately at a group of people who, perhaps as the result of discriminatory laws, are less likely to possess guns in the first place?

A few Second Amendment advocates on the political right, aligned with a handful of libertarians in the legal academy, have used the exposure of some gun laws’ racist provenance as a reason to insist that these laws should be abolished altogether. Clayton Cramer (2016), in an article funded by the National Rifle Association Institute for Legislative Action, has advanced this argument straightforwardly with respect to North Carolina’s pistol permit law. Cramer asserts that because the law was enacted a century ago as a tool to suppress the rights of black people, we should repeal the law today and allow everyone to buy handguns without a permit.

Similarly, with respect to ERPOs, a handful of sheriffs and county executives in several states have lately declared their gun-loving counties to be “Second Amendment sanctuaries” where ERPOs are best honored in the breach. Evidence of race-based differences in gun seizure practices elsewhere could only serve to confirm the sheriffs’ position. Along these lines, former Republican Senator Jim DeMint of South Carolina in 2019 penned an op-ed describing ERPOs as “great in theory, bad in practice.” DeMint cited a finding from our group’s research study in Indiana--the fact that many respondents failed to appear at a court hearing to reclaim their guns--and interpreted this as evidence that ERPOs discriminate against *“the less privileged … who cannot afford to take off work or who lack the means to hire good lawyers”* (DeMint 2019). If our initial published report had also included the fact that failure-to-appear was significantly more prevalent among nonwhite than white gun removal subjects in Indiana, Senator DeMint might have seized upon it to bolster his point that ERPOs can produce unfair results. But the disparate racial impact of ERPOs could cut both ways, with the result that families and communities of color could miss the benefits and protections of ERPOs, for example, as a suicide prevention tool (Swanson et al. 2017, 2019). Those who have experienced trauma and injustice in their dealings with police and the courts may never willingly turn to those same systems and trust them with something so sensitive as removing firearms from a loved one in a crisis.

On the pro-ERPO side are examples of ERPOs being used to deter would-be mass shooters (Wintemute et al. 2019) and thwart hate-fueled racist massacres (Carter 2020). Kaleb Cole, 24, was a suspected leader of the neo-Nazi group AtomWaffen Division and reportedly “preparing for a race war” in 2019. The FBI was tracking Cole and knew he had amassed a small arsenal of guns but could not charge him with a crime. Instead, the FBI collaborated with a specialized ERPO prosecutor unit in Seattle to seek an ERPO requiring Cole to surrender his firearms. Cole blatantly disregarded the ERPO and was later pulled over in Texas in a car with a Sig Sauer 9 mm pistol, an AR-15 rifle, two AK-47 rifles, and at least 1500 rounds of ammunition. Based on this information from another state, prosecutors in Seattle charged Cole with unlawful possession of firearms and issued a warrant for his arrest. He was later apprehended by the FBI.

Where ERPO supporters would applaud the use of these civil restraining orders to disarm and deter a dangerous neo-Nazi white nationalist from inciting a race war, ERPO detractors still fear the consequences of overreach. DeMint, in particular, cautioned that these “red flag laws” could be misused by “progressive judges” to abridge the Second Amendment rights of large categories of Americans considered to be a threat, including *“supporters of President Donald Trump, who according to some on the left are all racists and white nationalists.”* While perhaps rhetorically effective in building opposition to ERPOs from one side in our highly politicized national gun debate, DeMint’s argument essentially ignores research evidence that these laws can save lives (Cook and Donohue 2017; Swanson et al. 2017).

## Reconnecting ways of living and dying in the public health frame of gun violence

Saving lives matters. Striving to ensure that the lives saved are also respected--free from racial oppression, afforded equal justice--also matters. This means we cannot view gun violence (or research to understand it, or policies to prevent it) in isolation. We need to apply one of the oldest lessons of public health: ways of dying and ways of living are intimately connected. And in America, racial inequality, oppression, and injustice have significantly shaped that connection since the nation’s beginning.

On the mortality side of the equation, more than 100 people die every day from gunfire in the United States; every one of those deaths is a preventable tragedy that ripples through families and communities over generations. Black men under the age of 30 comprised 3% of the US population and 28% of the victims of firearm homicide in 2018. On the quality-of-life side of things, black people are incarcerated in state prisons at a rate 5 times higher than that of whites. Social determinants of violence and the disparate enforcement of gun laws both play a role in that deeply entrenched problem, but racial disparities are baked into the whole justice system. There are stark differences by race from one end to the other--from targeted policing, to profiling stops and arrests of minors, juvenile justice commitments, adult defendants’ access to bail and competent defense lawyers, referrals to treatment courts and jail diversion programs, harshness of prison sentences, parole decisions and the weight of coercive supervision in community corrections (Alexander 2010; Thompson 2019; Campbell and Schoenfeld 2013).

There is little doubt, then, that firearm restrictions predicated on criminal records are destined to reflect, in their targeting and enforcement, the imbalanced demographics of an unfair system in which those records are curated. As just one example, California has a law on the books that extends the minimum age for gun purchase to age 30 for former juvenile offenders (Harris 2016). The law seems to make perfect sense because it aligns with risk evidence and public health expert consensus (Webster and Vernick 2013). It also is virtually certain to affect young black men disproportionately, both in terms of exposure and compliance sanctioning. That much is unavoidable, insofar as the police, prosecutors, and judiciary have already functioned (unintentionally, one assumes) as a racial sorting machine. However, by the same token, the law could save many young black lives.

If our social pathologies and mortality rates involving racial inequities are linked, so must be our policy solutions and the research paradigms that evaluate and inform them. Public health, as an inherently interdisciplinary field spanning medicine, social sciences, and law, is well suited to lead the “war on disparities.” Nevertheless, action is urgent and need not wait for the experts in injury prevention and criminological policy to come to terms with each other (Cook and Ludwig 2019; Hemenway and Miller 2019). In the justice arena, for at least two decades we have had taskforce-produced, evidence-based, expert-approved, and stakeholder-blessed recommendations for thoroughgoing policy reforms to root out structural racism (e.g., The Sentencing Project, 2000; 2008; 2016). Some of these ideas--such as holding police more accountable for misconduct through weakening qualified immunity in the courts--are just now percolating through federal legislative proposals. Most of these measures should not even be controversial in 2020; they should have been approved and swiftly implemented long ago (Brennan Center for Justice 2020).

In times of social upheaval that call for action, existing reform coalitions often already know what to do. But they need to seize the new moment of broad-based community concern and engagement as rocket fuel for legislative action--for funding what works (and perhaps defunding what has miserably failed), commensurate with the evidence and the challenges they are all too aware of. Now is also the time for researchers to make the effort to incorporate robust measures of racial disparity impact into all of their empirical evaluations of these policies and institutional practices.

## Conclusion

Saving lives is not the only criterion for judging firearm laws’ effectiveness; the laws must also be equitable and fairly applied. What lies in the balance, and who should decide, especially when those concerned with risk vs. rights may come from opposite sides of a privilege chasm? While we struggle to resolve these tensions, there are concrete things to do that are not so complicated. To reiterate, we should surely make racial disparity impact a key outcome variable in our research into these laws’ effectiveness. We should also engage more deeply with affected communities, listen more carefully to their voices, incorporate their leadership in shaping more meaningful and authentic narratives to understand the tragedies of gun violence--in all of their human complexity--and thus find our way jointly to real and sustainable solutions. But here is our ongoing challenge: how can we best reconcile the worthy public health goals of saving lives through enacting and robustly enforcing evidence-based and constitutionally tested gun laws, with the moral imperative to stop the over-policing and mass incarceration of young men of color (Givens 2019)? The answers are not easy or readily forthcoming, but the persistent asking of such questions is finally being seen as urgent and essential.

## Data Availability

The dataset pertaining to gun seizure cases in Indiana that was analyzed during the current study is not publicly available due to IRB regulations. Upon request, the corresponding author will make available a de-identified data array for the two variables analyzed during the study (i.e., race and failure to appear in court).
