# Correction to: The color of risk protection orders: gun violence, gun laws, and racial justice

**DOI:** 10.1186/s40621-020-00274-x

**Published:** 2020-08-25

**Authors:** Jeffrey W. Swanson

**Affiliations:** 1 Department of Psychiatry and Behavioral Sciences, Duke University School of Medicine, Box 3071 Med Ctr, Durham, NC 27710 USA; 2 Brightleaf Square, Suite 23E, 905 W. Main St, Durham, NC USA

**Correction to: Inj Epidemiol 7, 46 (2020)**

**https://doi.org/10.1186/s40621-020-00272-z**

In the original publication of this article (Swanson, 2020), the author indicates that there are two errors in the second paragraph of the introduction. The corrections are below:

1. Inserting the word ‘insidious’ in the first sentence to stet the author’s original text.
2. Adding a ‘s’ to ‘invite’ in the last sentence so that the verb will match the singular subject of the sentence.

The publisher apologizes to the readers and authors for the inconvenience.

The original publication has been corrected.
